# Impact of Mass Drug Administration on Soil-Transmitted Helminth Infections Across Different Age Groups: A Retrospective Study From Southern Bihar

**DOI:** 10.7759/cureus.113764

**Published:** 2026-08-01

**Authors:** Kirti Vishwas, Ravindra K Barnawal, Ashwini Kumar, Anil C Phukan, Ranjan K Srivastava

**Affiliations:** 1 Microbiology, Narayan Medical College and Hospital, Sasaram, IND; 2 Microbiology, Autonomous State Medical College, Pilibhit, IND

**Keywords:** national deworming day, neglected tropical disease, soil-transmitted helminths, southern bihar, swachh bharat abhiyan

## Abstract

Background: Soil-transmitted helminth (STH) infections remain a major global public health concern, particularly in India. The Government of India has implemented mass drug administration (MDA) programs using albendazole to treat STH infections. However, the effectiveness of these deworming initiatives in reducing parasitic infections, particularly protozoal infections, remains incompletely characterized in many regions.

Methods: This retrospective observational study was conducted in the Department of Microbiology, Narayan Medical College and Hospital (NMCH), Sasaram, Bihar, India, from November 2023 to June 2025. The study included patients aged 1-84 years with documented prior albendazole deworming who underwent stool examination for suspected intestinal parasitic infections. Stool samples were processed using direct wet mount with saline and Lugol's iodine, and the salt flotation concentration method. Prevalence was calculated as frequencies and percentages. Fisher's exact test was used for comparisons among age groups using IBM SPSS Statistics version 28.0. Individual informed consent was waived, given the retrospective nature and use of anonymized laboratory records.

Results: Among 124 stool samples examined, 101 were included in the final analysis after excluding 23 samples for inadequate labeling, insufficient quantity, or missing demographic data. The mean age was 23.32 ± 5.42 years, with a male-to-female ratio of 1.59:1. Eight samples (7.9%) tested positive for parasitic infections: *Giardia lamblia* (n=6, 5.9%) and *Entamoeba histolytica* (n=2, 2.0%). No STH ova were detected. Ninety-three samples (92.1%) tested negative. Age-stratified analysis showed the highest parasitic prevalence in school-aged children (17.65%), followed by preschool-aged children (15.0%), with lower prevalence in adults (5.12%) and no infections in adolescents or the elderly. Fisher's exact test revealed no statistically significant difference in parasitic infection prevalence across age groups (P=0.175).

Conclusion: The low prevalence of STH reflects the effectiveness of national MDA efforts and improved sanitation in southern Bihar. However, persistent protozoal infections despite prior deworming highlight the need for comprehensive water, sanitation, and hygiene (WASH) interventions and hygiene education to address protozoal transmission pathways not controlled by albendazole-based deworming programs.

## Introduction

Soil-transmitted helminths (STH), including roundworm (*Ascaris lumbricoides*), whipworm (*Trichuris trichiura*), and hookworms (*Ancylostoma duodenale* or *Necator americanus*), are common human infections. Approximately 1.45 billion people worldwide are affected, with India accounting for 21% of cases [1].

The Government of India (GOI) has implemented deworming programs focusing initially on children, now extended to adolescents up to 19 years and women aged 15-49 years. Albendazole is used in these initiatives and effectively treats STH infections with a single dose [2].

In developing countries, both urban and rural populations bear a significant burden of protozoal and helminthic infections [3]. Several predisposing factors, such as inadequate drinking water, substandard sanitation, and poor hand and body hygiene, can lead to parasitic infections [4]. Some common consequences of intestinal parasitic infections (IPIs) in children include impaired school performance, easy fatigability, stunted growth, and deficiencies in iron and other nutrients [5]. The *Entamoeba histolytica* and *Giardia duodenalis/Giardia intestinalis *are the most commonly isolated intestinal protozoa in humans. Helminths, such as roundworm (*Ascaris lumbricoides*), whipworm (*Trichuris trichiura)*, and hookworms (*Necator americanus* and *Ancylostoma duodenale*), are predominantly STH. Roundworm and whipworm mainly affect children, whereas hookworm affects both children and young adults. Therefore, early preventive measures are crucial for eradicating STH by understanding their epidemiology and clinical features [6].

In September 2004, the World Health Organization (WHO) included Giardiasis in the Neglected Disease Initiative due to its impact on pregnant women and children and its link to poverty. *G. duodenalis *affects 300 million people in Africa, Asia, and Latin America, mainly children in low-income areas [7]. The infection rate is estimated at 20-30% in developing countries and 2-5% in industrialized nations [8].

Globally, STH are the most common helminthic infections, affecting approximately 1.22 billion people worldwide [9]. The prevalence of STH in Asian countries is 67%, with India having the highest prevalence at 21%, followed by China at 18% [6]. In India, around 225 million children are at risk of STH, and 39 million disability-adjusted life years are attributed to intestinal parasitic infections, resulting in a significant economic burden [10].

The WHO asserts that preventive chemotherapy, involving the periodic large-scale administration of anthelminthic agents to at-risk populations, can significantly reduce the burden of worms caused by STH infections. Reducing the worm burden of STH helps to decrease morbidity among individuals heavily infected by these helminths.

Since preventive chemotherapy does not interrupt the cycle of infection and reinfection, populations residing in contaminated environments remain at risk and require frequent administrations of anthelminthic medications. Given the relationship between the prevalence and intensity of STH infections, only light-intensity infection and low morbidity are anticipated where the baseline prevalence of any STH infection is below 20%.

As a public health intervention, WHO recommends preventive deworming with a single dose of albendazole (400 mg) or mebendazole (500 mg), administered annually or biannually, for all young children (12-23 months of age, albendazole 200 mg), preschool-aged children (24-59 months of age), and school-aged children living in areas where the baseline prevalence of any soil-transmitted infection is 20% or higher among children, to mitigate the worm burden of STH infections [11].

In India, 241 million children need deworming. Bihar, a densely populated state with low development indicators, highlights this issue. The Swachh Bharat Abhiyan (Clean India Mission) was a national campaign from 2014 to 2019 to clean up India's streets, roads, and infrastructure. Its goal was to eliminate open defecation by building household and community toilets and monitoring their use. By October 2, 2019, on the 150th birth anniversary of Mahatma Gandhi, the mission achieved an open defecation-free status for India by constructing 90 million toilets in rural areas at a cost of 1.96 lakh crores (US$ 30 billion) [12].

On February 10, 2015, India's Ministry of Health and Family Welfare (MOHFW) launched the first National Deworming Day (NDD), targeting nearly 241 million children in 12 states to combat STH infection. This initiative aimed to reduce absenteeism in schools, improve health and learning outcomes, and increase chances of higher-wage jobs [13]. Furthermore, the global acceptance of a single-dose regimen has been considered both safe and advantageous. Albendazole tablets are recognized worldwide as an evidence-based, effective solution for controlling worm infections [14].

Study objectives

The study aimed to determine the current prevalence of intestinal parasitic infections in a patient population with a documented history of prior deworming who presented to a tertiary care hospital in southern Bihar, India, and to characterize the epidemiological patterns of parasitic infections in this setting [15].

The secondary objectives were to describe the age-specific prevalence of parasitic infections across the study population, identify the types and frequencies of parasitic organisms detected, assess gender-based differences in the prevalence of parasitic infections, characterize the temporal distribution of parasitic infections during the study period, and provide descriptive data on the current burden of parasitic infections in a tertiary care setting serving both urban and peri-urban populations.

## Materials and methods

Study design and setting

This retrospective observational study was conducted in the Department of Microbiology at Narayan Medical College and Hospital (NMCH), Sasaram, Bihar, India. The study period extended from November 2023 to June 2025, covering 20 months.

Study population and eligibility criteria

The study population comprised patients aged 1-84 years who presented to various clinical departments. The primary inclusion criterion was having undergone stool examination for suspected intestinal parasitic infection and having a documented history of prior albendazole deworming.

Patients were categorized into predefined age groups for analytical purposes: preschool-aged children (1-4 years), school-aged children (5-14 years), adolescents and young adults (15-24 years), adults (25-59 years), and older adults (≥60 years). According to WHO guidelines for preventive deworming, children aged <24 months receive a single 200-mg dose of albendazole, whereas those aged ≥24 months, adolescents, and adults receive a single 400-mg dose. Although all study participants had a documented history of prior albendazole deworming, the specific dose and timing of administration were not systematically recorded, limiting the ability to correlate these factors with parasitic infection outcomes.

The exclusion criteria included inadequately labeled or insufficient stool samples, lack of essential demographic information, and stool samples submitted for non-parasitic investigations.

Sample collection and processing

Stool samples were collected in sterile, preservative-free containers and processed within two to three hours of collection to ensure optimal diagnostic yield. Comprehensive macroscopic and microscopic examinations were performed. Macroscopic evaluation assessed the samples for color, consistency, and the presence of blood, mucus, or adult worms.

Microscopic examination was conducted using direct wet-mount preparations (saline and Lugol’s iodine) and the salt flotation concentration method to enhance the detection of parasitic ova and cysts. Parasites were systematically identified using both low-power and high-power microscopy. The prevalence of parasitic infections was determined by calculating the percentage of processed samples in which parasites were detected.

The temporal distribution of sample collection revealed notable seasonal variation over the 20-month study period (November 2023 to June 2025). Specifically, 33.66% of the samples (n = 34) were collected during the monsoon season (June-July 2024). This seasonal peak may reflect an increased incidence of parasitic infections during periods of elevated environmental moisture and potential water contamination. Active sample collection was maintained from November 2023 through January 2025, with no additional samples collected during the final five months of the study period (February-June 2025).

Data collection and statistical analysis

Relevant sociodemographic, epidemiological, and behavioral data, including sanitation practices and deworming history, were retrospectively extracted using an unstructured questionnaire. All collected data were systematically recorded in a Microsoft Excel spreadsheet (Microsoft Corporation, Redmond, Washington).

Statistical analysis was performed using IBM SPSS Statistics for Windows, Version 28 (Released 2021; IBM Corp., Armonk, New York). Descriptive statistics were presented as frequencies, proportions, and percentages. The Fisher's exact test was employed to analyze the distribution of study age groups and assess the statistical significance of quantitative data associations. A p-value of less than 0.05 was considered statistically significant.

Ethical considerations

The study protocol was reviewed and approved by the Departmental Research Committee of the Department of Microbiology, NMCH, Sasaram, Bihar (approval number: NMCH/Micro/26/128; date: June 27, 2026). A waiver of informed consent was granted for this retrospective observational study because the research involved no more than minimal risk, used only existing anonymized laboratory records and patient data collected during routine clinical care, involved no direct patient contact or intervention, and maintained participant privacy and confidentiality. The study was conducted in accordance with the Indian Council of Medical Research (ICMR) National Ethical Guidelines for Biomedical and Health Research Involving Human Participants (2017) and applicable institutional policies.

## Results

Demographic characteristics

A total of 124 stool samples were initially received for parasitological examination. After the application of the exclusion criteria, 23 samples were excluded because of inadequate labeling (n = 12), insufficient sample quantity (n = 7), lack of essential demographic data (n = 3), or a processing issue identified during the final review (n = 1). The final study population comprised 101 patients.

The demographic characteristics of the study population are presented in Table 1. The mean age of the study participants was 23.32 ± 5.42 years. The age range was broad, spanning from 1 to 84 years, allowing for a comprehensive life-course assessment of parasitic infection prevalence. The male-to-female ratio was 1.59:1, with males accounting for 61.4% (n = 62) of the study population and females accounting for 38.6% (n = 39). 

**Table 1:** Demographic characteristics and age distribution of study participants Values are presented as mean ± standard deviation (SD) The study population comprised 101 participants with a male-to-female ratio of approximately 1.59:1. Age data were analyzed across all study participants aged 1-84 years presenting with suspected intestinal parasitic infections and prior albendazole deworming history. Statistical analysis was performed using IBM SPSS Statistics for Windows, Version 28 (Released 2021; IBM Corp., Armonk, New York). Descriptive statistics included the calculation of means and standard deviations for continuous variables.

Demographic Characteristic	Male (n = 62)	Female (n = 39)	Overall (n = 101)
Age (years), Mean ± SD	24.9 ± 3.8	20.8 ± 3.8	23.32 ± 5.42

Age group distribution

The study population was stratified into five predefined age groups for comparative analysis, as detailed in Table 2. The adult age group (25-59 years) constituted the largest segment of the cohort, accounting for 38.6% (n = 39). This was followed by preschool-aged children (1-4 years) at 19.8% (n = 20), school-aged children (5-14 years) at 16.8% (n = 17), elderly participants (≥60 years) at 13.9% (n = 14), and adolescents and young adults (15-24 years) at 10.9% (n = 11). Statistical analysis using Fisher’s exact test revealed a statistically significant difference in the distribution of age groups between males and females (p = 0.0019). Younger age groups showed a female predominance, while older age groups showed a male predominance. 

**Table 2:** Age group distribution of study participants based on gender Data are presented as numbers (n) with percentages in parentheses. Row percentages indicate the proportion within each gender group; column percentages indicate the proportion within each age group. Fisher's exact test revealed a statistically significant difference in gender distribution across age groups (p = 0.0019). The study population comprised 101 patients aged 1-84 years from various clinical departments with a documented history of prior albendazole deworming and suspected intestinal parasitic infections. Statistical analysis was conducted using IBM SPSS Statistics for Windows, Version 28 (Released 2021; IBM Corp., Armonk, New York). A p-value of <0.05 was considered statistically significant.

Gender	Preschool-Aged (1-4 Years)	School-Aged (5-14 Years)	Adolescents and Young Adults (15-24 years)	Adults (25-59 years)	Elderly (60+ years)	Total
Male	9 (45%)	8 (47.1%)	3 (27.3%)	31 (77.5%)	11 (78.6%)	62 (61.4%)
Female	11 (55.0%)	9 (52.9%)	8 (72.7%)	8 (20.0%)	3 (21.4%)	39 (38.6%)
Total	20 (19.8%)	17 (16.8%)	11 (10.9%)	39 (38.6%)	14 (13.9%)	101 (100%)

Temporal distribution of sample collection

During the 20-month study period from November 2023 to June 2025, active sample collection occurred primarily from November 2023 to January 2025. The temporal distribution of sample collection demonstrated marked seasonal variation. The highest collection rates were observed during the monsoon season, specifically in June and July 2024, which together accounted for 33.66% (n = 34) of all samples. This peak correlates with the recognized epidemiological pattern of increased gastrointestinal presentations during periods of high humidity and rainfall, which are known to facilitate the environmental survival and transmission of parasitic organisms. Conversely, lower collection rates were observed during the winter and early summer months.

Stool examination results and parasitic pathogen distribution

Overall Prevalence of Parasitic Infections

**Figure 1:**
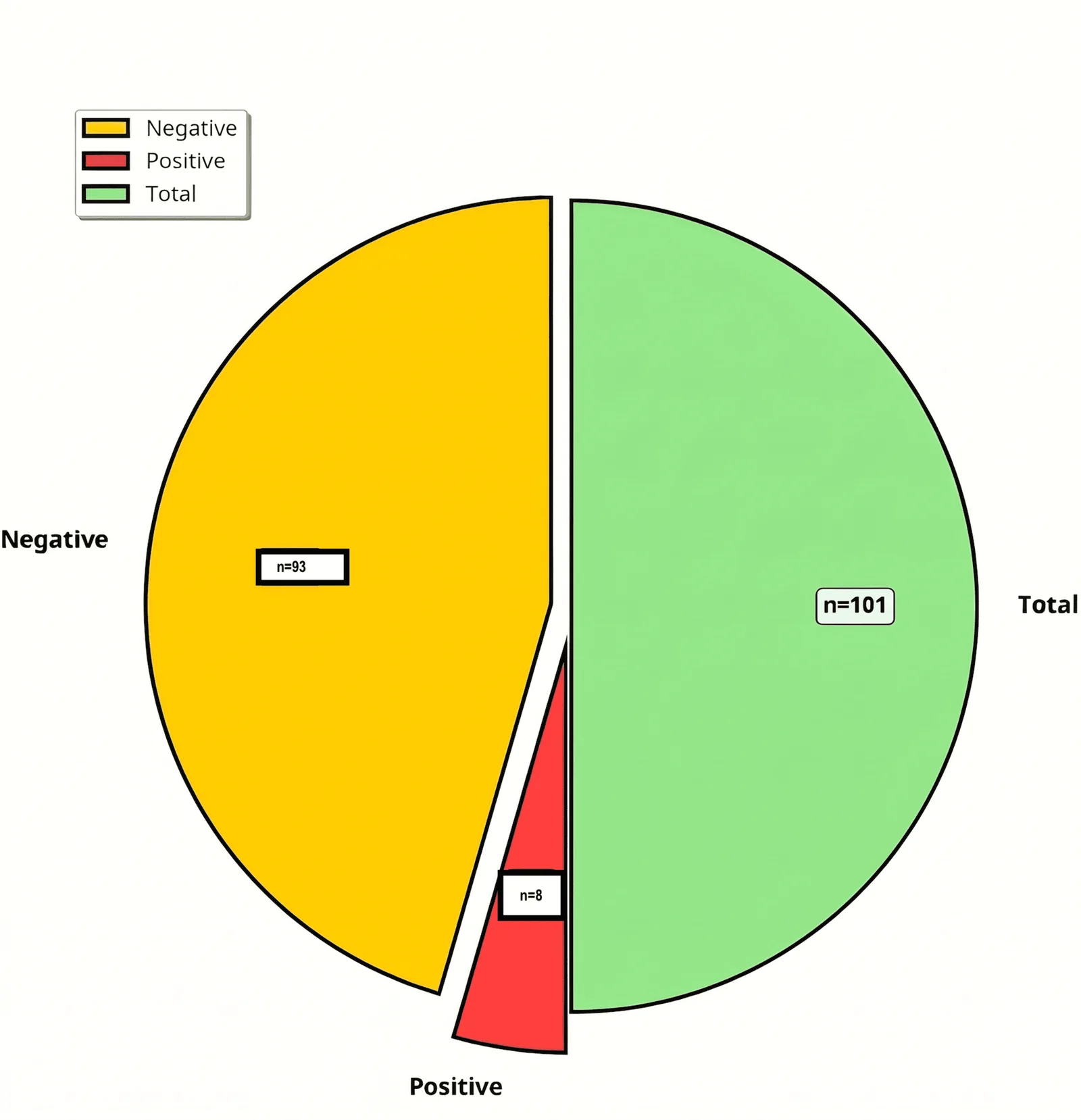
Distribution of stool examination results The figure illustrates the distribution of stool examination results among the 101 patients included in the study. Of the total samples examined, 93 samples (92.08%) were negative for parasitic infections, while 8 samples (7.92%) tested positive for parasitic or fungal organisms. The negative samples are represented in gold, positive samples are represented in red and total sample represented as green. The pie chart provides a visual representation of the overall prevalence of parasitic infections in the study population, demonstrating that the majority of participants had negative stool examination results despite prior albendazole deworming history. The positive samples identified *Giardia lamblia* (n = 6, 5.94%) and *Entamoeba histolytica *(n = 2, 1.98%). The predominance of negative results reflects the effectiveness of national deworming programs and improvements in sanitation infrastructure in the study region.

Of the 101 stool samples examined, the vast majority (92.08%, n = 93) tested negative for any parasitic infections. Only eight samples (7.92%) tested positive for parasitic organisms, yielding an overall parasitic infection prevalence of 7.92% in this population with a documented history of prior deworming. The distribution of these stool examination results is visually summarized in Figure 1.

Identified Parasitic Pathogens

Among the eight positive samples, two distinct parasitic organisms were identified through microscopic examination (Table 3). The protozoan Giardia lamblia was the predominant pathogen, detected in 75.0% (n=6) of the positive samples and representing a prevalence of 5.94% in the total study population. Entamoeba histolytica, another protozoan, was identified in 25.0% (n = 2) of the positive samples (1.98% of the total population). Crucially, comprehensive microscopic examination using both direct wet mounts and the salt flotation concentration method revealed a complete absence of helminth ova in all 101 samples examined. This finding indicates a zero prevalence of STH infections in this specific study cohort. 

**Table 3:** Identified parasitic pathogens from positive samples (n = 8) Among the 101 stool samples examined, 8 (7.92%) were positive for parasitic organisms. *Giardia lamblia* was the predominant organism, accounting for more than two-thirds of all positive cases. *Giardia lamblia* is a protozoan parasite that causes giardiasis, a common intestinal infection associated with poor sanitation and contaminated water sources. The detection rate of 5.94% in this study is consistent with the epidemiology of parasitic infections in endemic regions. *Entamoeba histolytica*, another protozoan parasite, was identified in 1.98% of the study population. This organism is associated with amebic dysentery and liver abscess formation in severe cases. The prevalence of parasitic infections in this study (7.92%) reflects the burden of intestinal parasitic diseases in the study population, which may be influenced by factors such as sanitation practices, access to clean water, and prior deworming history. All parasitic organisms were identified using standard parasitological examination techniques, including direct wet-mount preparations and salt flotation concentration methods, with confirmation under both low-power and high-power microscopy. Statistical analysis was conducted using IBM SPSS Statistics for Windows, Version 28 (Released 2021; IBM Corp., Armonk, New York).

Parasitic Pathogen	Number (n)	Percentage (%)	Age Groups Affected
Giardia lamblia	6	5.94	Preschool-aged (n = 2), School-aged (n = 2), Adults (n = 2)
Entamoeba histolytica	2	1.98	Preschool-aged (n = 1), School-aged (n = 1)
Total	8	7.92	—

Age-Specific Prevalence of Parasitic Infections

The distribution of stool examination results across the predefined age groups revealed distinct epidemiological patterns (Table 4). Parasitic infections were disproportionately concentrated in the younger age cohorts. School-aged children (5-14 years) demonstrated the highest age-specific prevalence at 17.65% (3/17). This was closely followed by preschool-aged children (1-4 years), who exhibited a prevalence of 15.0% (3/20).

**Table 4:** Stool examination results and age-specific prevalence of parasitic infections Data are presented as the number (n) of participants with percentages calculated as (positive cases/total participants in the age group) × 100. Statistical analysis using Fisher's exact test revealed no statistically significant difference in parasitic infection prevalence across age groups (p = 0.175). Identification and distribution of parasitic organisms detected in positive stool samples, stratified by age group. Among the 8 positive samples for parasitic infections, two parasitic organisms were identified: *Giardia lamblia *(n = 6, 5.94% of total population, 75.0% of positive samples) and *Entamoeba histolytica *(n = 2, 1.98% of total population, 25.0% of positive samples). *Giardia lamblia *was detected across three age groups: preschool-aged children (n = 2), school-aged children (n = 2), and adults (n = 2). *Entamoeba histolytica *was detected exclusively in younger age groups: preschool-aged children (n = 1) and school-aged children (n = 1). The overall parasitic infection prevalence in the study population was 7.92% (8/101). *Candida*/yeast cells, which are fungi and not parasitic organisms, have been appropriately excluded from this analysis. One *Candida* case was isolated from an adult patient and represents a separate microbiological finding not related to parasitic infection burden. All samples were examined using microscopic methods, including direct wet mount with saline and Lugol's iodine, and the salt flotation concentration technique. Statistical analysis was conducted using IBM SPSS Statistics for Windows, Version 28 (Released 2021; IBM Corp., Armonk, New York). A p-value of <0.05 was considered statistically significant.

Age Group	Negative (n)	Positive (n)	Total (n)	Prevalence (%)
Preschool-aged (1-4 years)	17	3	20	15.0
School-aged (5-14 years)	14	3	17	17.65
Adolescents and young adults (15-24 years)	11	0	11	0
Adults (25-59 years)	37	2	39	5.12
Elderly (60+ years)	14	0	14	0
Total	93	8	101	7.92

In contrast, no parasitic infections were detected in the adolescent and young adult group (15-24 years) or the elderly group (≥60 years). The adult group (25-59 years) demonstrated an intermediate prevalence of 5.12% (2/39). Despite these observable differences in infection rates across age strata, Fisher’s exact test revealed that these variations did not reach statistical significance (p = 0.175). This lack of statistical significance may be attributed to the relatively small number of positive cases overall and the constrained sample sizes within individual age groups.

All participants aged ≥15 years would have received the standard 400-mg albendazole dose according to WHO guidelines for preventive deworming. In contrast, preschool-aged children (1-4 years), who may have received a lower dose (200 mg if aged under 24 months) according to WHO recommendations, demonstrated a higher infection prevalence of 15.0% (3/20), suggesting that age-appropriate dosing considerations warrant further investigation in future studies. 

Clinical significance and deworming history

All participants in this study had a documented history of prior albendazole deworming. In this context, the complete absence of helminthic infections aligns with the established efficacy of albendazole as a broad-spectrum anthelmintic agent. However, the detection of an overall parasitic infection prevalence of 7.92%, driven entirely by protozoan organisms (*Giardia lamblia *and *Entamoeba histolytica*), underscores a critical clinical reality. Standard MDA protocols using albendazole are highly effective against targeted helminths but offer no protection against protozoal infections. The persistence of these protozoal pathogens, particularly in preschool- and school-aged children, highlights the limitations of pharmacologic deworming alone and indicates ongoing exposure to contaminated water or food sources, necessitating complementary public health interventions.

The retrospective analysis of 101 stool samples revealed an overall parasitic infection prevalence of 7.92%, with *Giardia lamblia *being the most frequently detected organism (5.94%). Age-specific analysis demonstrated higher infection rates in younger age groups, particularly school-aged children (17.65%) and preschool-aged children (15.0%), although these differences were not statistically significant. The absence of helminthic infections despite prior deworming suggests effective anthelmintic coverage in the study population, while the detection of protozoal parasites highlights the need for targeted interventions against protozoan infections in endemic regions.

## Discussion

Parasitic infection prevalence and public health implications

The absence of STH infections and the low protozoan positivity (7.92%) in this study suggest significant progress in STH control, likely due to MDA campaigns and improved sanitation in southern Bihar. In our study, preschool-aged children (1-4 years) showed a parasitic infection prevalence of 15.0% (3/20), while school-aged children (5-14 years) demonstrated a prevalence of 17.65% (3/17). These findings are consistent with the known epidemiology of intestinal parasitic infections, which disproportionately affect younger populations due to factors such as developing immune systems, frequent contact with contaminated soil or water during play, and suboptimal personal hygiene practices.

The complete absence of STH infections in our study population, all of whom had a documented history of prior albendazole deworming, provides compelling evidence for the efficacy of current MDA programs in this region. This finding aligns with the primary goal of the NDD initiative, which aims to reduce the STH burden in children and adolescents. The zero prevalence of STH suggests that the periodic administration of albendazole is successfully interrupting the transmission cycle of these helminths or maintaining the worm burden at undetectable levels in the studied cohort.

National deworming and sanitation initiatives

The NDD, launched in 2015, targeted 241 million children and was later expanded to include adolescents and reproductive-aged women [13]. In parallel, the Swachh Bharat Abhiyan (Clean India Mission) dramatically increased access to sanitation by constructing over 90 million toilets [12]. Southern Bihar, including Sasaram, has witnessed tangible improvements in sanitation coverage, latrine use, and hygiene awareness. In our study, environmental improvements such as access to treated water, household latrines, and a reduction in open defecation have disrupted the fecal-oral transmission cycle, contributing to reduced STH prevalence.

In contrast to our findings, earlier studies such as Steinmann et al. reported a significantly higher intestinal parasitic infection (IPI) prevalence of 40.7%, especially among school-aged children [14]. Similarly, Ajjampur et al. found a 17% prevalence of STHs in southern India [15]. These differences may be attributed to regional demographic variations, improved public health measures, or the effective implementation of government-led deworming campaigns in our study area. The low prevalence among children (both preschool- and school-aged) supports the continued use of albendazole and reinforces its safety and efficacy in mass campaigns [16].

The WHO’s 2021-2030 Neglected Tropical Diseases (NTD) Roadmap focuses on eliminating STH morbidity among preschool-aged children, school-aged children, and reproductive-aged women [17]. Our findings demonstrate progress toward this ambitious goal in the study region, although continued vigilance remains necessary.

Sample size and study design considerations

The overall sample size of 101 stool samples may appear modest; however, it is important to note that the study was designed to reflect a broad spectrum of age groups, ranging from 1 to 84 years, rather than focusing on a single cohort such as school-aged children (5-14 years). This diversity allowed for an age-stratified assessment of parasitic infections, offering practical insights into the epidemiological transition and impact of deworming across the life course. Our study cohort had a mean age of 23.32 ± 5.42 years, covering a wide range of age groups, including those targeted by national deworming efforts (Table 1). Only eight of the 101 stool samples tested positive for intestinal parasites, with no STHs detected, highlighting the potential effectiveness of both pharmacologic (MDA) and nonpharmacologic (sanitation) strategies.

Protozoan infections and hygiene challenges

Among protozoan infections, *Giardia lamblia* was the most prevalent organism, detected in 5.94% of the study population, followed by *Entamoeba histolytica* at 1.98% (Table 3). Although these infections were mostly self-limiting, their presence indicates the need for continued attention to hygiene and water sanitation, especially in vulnerable populations. The detection of protozoal infections such as *Giardia lamblia* and *Entamoeba histolytica* points to continued challenges related to water contamination, inadequate handwashing, and community hygiene practices. Thus, deworming must be complemented by Water, Sanitation, and Hygiene (WASH) interventions to achieve long-term control of parasitic infections.

Albendazole efficacy and age-specific patterns

Albendazole has been widely used in mass deworming campaigns under the Government of India’s national programs. Efficacy studies conducted across multiple countries, including India, have confirmed that even a single dose of albendazole is highly effective in reducing the STH burden. According to WHO guidelines, albendazole dosing for preventive deworming is age-dependent: children under 24 months receive a single dose of 200 mg, while children aged 24 months and older receive a single dose of 400 mg.

The age-dependent dosing strategy reflects differences in pharmacokinetics and safety considerations across age groups. In our study, patients in the adolescent and young adult age group (15-24 years) and the elderly age group (≥60 years), who would have received the standard 400-mg dose according to WHO guidelines, showed no positive stool samples. Only three of the 40 samples from the adult age group (25-59 years) were positive, indicating a prevalence of 7.5%. In contrast, preschool-aged children (1-4 years) demonstrated a higher infection prevalence of 15.0% (3/20), which may reflect the lower albendazole dose (200 mg) recommended by WHO for children under 24 months or, alternatively, increased exposure to parasitic infections in younger age groups due to behavioral and environmental factors.

Diagnostic limitations and interpretation

The complete absence of helminth ova must be interpreted cautiously. While it may reflect the success of sustained mass drug administration (MDH) and improved sanitation indicators, diagnostic limitations also need to be considered. A single stool sample and saline/iodine mounts have limited sensitivity for low-intensity STH infections. The WHO recommends at least two stool samples and Kato-Katz or molecular methods to detect low-grade transmission [17]. Therefore, our findings should be interpreted as an encouraging trend rather than definitive elimination.

Temporal and seasonal patterns

In our study, 101 samples were collected between November 2023 and June 2025, with the highest numbers of samples collected in July 2024 (n=18) and June 2024 (n=16), aligning with the monsoon season in Bihar. This is of particular relevance, as STH transmission is known to be influenced by climatic conditions such as humidity, temperature, and soil moisture, all of which are elevated during the monsoon. These environmental factors enhance the survival and infectivity of helminth eggs and larvae in the soil, potentially increasing the risk of transmission. Conversely, fewer samples were collected during the winter months (e.g., December 2023 and 2024), which might correlate with lower environmental viability of helminths due to cooler and drier conditions. Analyzing stool samples across different seasons-winter (December-February), summer (March-May), monsoon (June-September), and post-monsoon/autumn (October-November)-enables a more comprehensive understanding of STH epidemiology in this region. This temporal surveillance can aid in optimizing the timing of mass deworming programs and targeted interventions based on high-risk seasons.

Global context and regional significance

Globally, STH infections affect approximately 27% of preschool- and school-aged children [18]. The markedly low prevalence in our setting supports the success of periodic mass deworming, particularly when combined with improved sanitation and awareness campaigns. It also highlights the reduced need for routine deworming in certain urban populations, although peri-urban and underserved urban regions, particularly in eastern Bihar, may still be at increased risk due to rapid urbanization.

The observed low prevalence of STHs and the absence of helminth ova in this study population may be reflective of broader improvements in public health and sanitation indicators in southern Bihar, particularly in Rohtas district. According to the National Family Health Survey (NFHS-5, 2019-2021), the state has shown improvements in key health determinants such as access to improved sanitation facilities (from 25.2% in NFHS-4 to 62.8% in NFHS-5) and toilet use (households with access to sanitation facilities increased from 37% to 70%) [19].

Age-dependent albendazole dosing and infection prevalence

The WHO recommends age-dependent dosing of albendazole for preventive deworming programs, with children under 24 months receiving 200 mg and older children, adolescents, and adults receiving 400 mg. This dosing strategy is based on pharmacokinetic studies demonstrating that younger children have different drug absorption and metabolism patterns compared with older children and adults [20].

While the specific albendazole doses administered to individual participants in this study were not recorded, the observed age-specific infection prevalence patterns may reflect the impact of age-dependent dosing regimens. The higher infection rate observed in preschool-aged children (15.0%) compared with older age groups (0-7.5%) could potentially be attributed to the lower 200-mg dose recommended for children under 24 months by WHO guidelines [21]. However, this interpretation must be considered cautiously, as multiple factors contribute to parasitic infection risk in younger children, including increased exposure through behavioral factors (hand-to-mouth contact and playing in soil), dietary habits, and immune system maturation. Future prospective studies that systematically record albendazole dosing, timing of administration, and the interval since the last deworming would be valuable in determining whether age-dependent dosing variations contribute to the observed differences in infection prevalence across age groups.

## Conclusions

The low prevalence of STH reflects the effectiveness of national MDA efforts and improved sanitation in southern Bihar. The observed age-specific infection patterns, with higher prevalence in younger age groups despite prior deworming, highlight the need for comprehensive WASH interventions and hygiene education to address protozoal transmission pathways not controlled by albendazole-based deworming programs.
